# Impact of sarcopenia on ventricular remodelling following coronary artery bypass grafting in elderly patients with coronary heart disease

**DOI:** 10.3389/fcvm.2025.1635863

**Published:** 2026-04-29

**Authors:** Hongfang Li, Fangfang Ma, Yite Li, Zhenyu Su, Xugang Wang

**Affiliations:** 1 Department of Cardiac Surgery, The First Hospital of Hebei Medical University, Shijiazhuang, Hebei, China; 2 Department of Cardiology, The First Hospital of Hebei Medical University, Shijiazhuang, Hebei, China; 3 Department of Cardiac Surgery, Hebei Medical University, Shijiazhuang, Hebei, China

**Keywords:** coronary heart disease, elderly patients, coronary artery bypass grafting, left ventricular remodelling, sarcopenia

## Abstract

**Objective:**

To investigate the association between sarcopenia and ventricular remodelling after coronary artery bypass grafting (CABG) in elderly patients with coronary heart disease (CHD).

**Methods:**

A total of 135 elderly patients with CHD admitted to hospital between February 2021 and May 2023 were prospectively selected. According to whether ventricular remodelling occurred during the follow-up period, patients were divided into an occurrence group and a non-occurrence group.

**Results:**

The incidence of ventricular remodelling after CABG in elderly patients with CHD was 23.70% (32/135). Significant differences between the two groups were observed in smoking history, diabetes history, prevalence of sarcopenia, degree of preoperative coronary stenosis, levels of lipoprotein (a) ([Lp(a)]), uric acid (UA), protease-activated receptor 2 (PAR2), monocyte-to-HDL ratio (MHR), and suspended red blood cell input (*P* < 0.05). Multivariable logistic regression analysis identified smoking history [odds ratio (OR) = 2.186, 95% confidence interval (CI) 1.34–3.57], diabetes history (OR = 2.171, 95% CI 1.32–3.58), sarcopenia (OR = 2.230, 95% CI 1.37–3.62), a high degree of preoperative coronary stenosis (OR = 2.223, 95% CI 1.36–3.64), elevated Lp(a) (OR = 2.143, 95% CI 1.32–3.49), elevated UA (OR = 2.164, 95% CI 1.31–3.58), elevated PAR2 (OR = 2.192, 95% CI 1.32–3.64), and elevated MHR (OR = 2.201, 95% CI 1.32–3.68) as independent risk factors for ventricular remodelling after CABG in the study population. Analysis showed that sarcopenia predicted ventricular remodelling with a sensitivity of 90.0%, specificity of 47.0%, and an area under the curve of 0.772 (95% CI 0.68–0.86, *P* < 0.0001).

**Conclusion:**

Sarcopenia is a risk factor affecting ventricular remodelling after CABG in elderly patients and shows strong predictive efficacy in this population.

## 1 Introduction

With the ageing of modern society, the incidence of coronary heart disease (CHD) in the elderly population continues to rise each year. The disease remains incurable, and current clinical approaches primarily aim to relieve symptoms and prolong survival through medication, interventional surgery, and coronary artery bypass grafting (CABG) (1, 2).

Percutaneous coronary intervention is widely used in the treatment of CHD in the elderly due to its advantages of minimal trauma, rapid postoperative recovery, and high safety. Conversely, CABG is the preferred option for more serious CHD, such as stenosis of 70% or more of the coronary artery trunk and branches, myocardial infarction accompanied by ventricular aneurysm, or valvular closure insufficiency (3, 4).

Coronary artery bypass grafting is an effective clinical revascularisation technique for severe coronary lesions, and with continuous improvements in surgical quality, the survival rate of elderly patients has increased considerably (5). However, oedema and necrosis of cardiomyocytes may occur secondary to CABG in elderly patients with CHD, further altering endocrine regulation, neuromodulation, and local myocardial motion and function. These pathological changes ultimately lead to ventricular remodelling and compromise long-term prognosis (6). Early identification and control of risk factors affecting ventricular remodelling are, therefore, crucial for improving outcomes in this population.

Research has found that a decline in skeletal muscle quality, strength, or function is associated with a poor prognosis (7). Sarcopenia, defined as a syndrome characterised by generalised muscle loss, decreased muscle strength, and impaired physiological function, is a major contributor to cardiopulmonary decline and bone fractures in the elderly (8). The co-morbidity of CHD and sarcopenia is relatively common: CHD can promote sarcopenia development through oxidative stress, autophagy, apoptosis, and inflammation, and sarcopenia, in turn, affects cardiovascular prognosis by impairing muscle strength and metabolic regulation (9–11). Recent studies have shown that patients with concurrent sarcopenia have a higher incidence of major adverse cardiac and cerebral events and shorter survival after CABG (12). Moreover, skeletal muscle deterioration may aggravate insulin resistance and atherosclerosis or interact with neurohormonal and inflammatory pathways, thereby increasing the risk of ventricular remodelling after CABG (13, 14).

Previous research has linked sarcopenia or frailty to mortality, morbidity, and delayed recovery after cardiac procedures. However, whether sarcopenia independently predicts ventricular remodelling after CABG and how it integrates with inflammatory–metabolic markers to improve risk discrimination, remains insufficiently defined, particularly within Asian cohorts.

The present study prospectively evaluates elderly Chinese patients undergoing CABG and focuses on postoperative ventricular remodelling as the primary outcome (change in left ventricular end-diastolic volume [ΔLVEDV] ≥ 15%). In addition to sarcopenia, protease-activated receptor 2 (PAR2) and the monocyte-to-HDL ratio (MHR) are examined as adjunct markers that may capture inflammation- and metabolism-related risk pathways implicated in remodelling. By reporting both discrimination (receiver operating characteristic [ROC]/area under the curve [AUC]) and calibration (Hosmer–Lemeshow test, calibration intercept/slope), the aim is to provide transparent, clinically interpretable model performance and a theoretical basis for better clinical guidance of post-CABG rehabilitation in elderly patients with CHD, as reported below.

### 1.1 Information and methods

#### 1.1.1 Sample size justification

Before enrolment, an *a priori* power calculation for the primary endpoint (post-CABG ventricular remodelling, defined as ΔLVEDV ≥ 15%) was performed. Assuming a remodelling incidence of 30% in the overall cohort, an expected risk increase associated with sarcopenia corresponding to an odds ratio (OR) = 2.0, two-sided *α* = 0.05, and 80% power, the minimum required sample size was approximately 128 participants to provide a balanced and effective sample for logistic regression. To allow for potential loss to follow-up and measurement uncertainty, the target sample size was set at ≥130 participants. The final cohort included 135 patients, meeting the prespecified power requirement.

#### 1.1.2 General information

A total of 135 elderly patients with CHD who were admitted to hospital between February 2021 and May 2023 were prospectively selected. The inclusion criteria were as follows: (1) age ≥60 years, (2) diagnosis of CHD confirmed by coronary angiography, (3) fulfilment of surgical indications for CABG and successful completion of the procedure, (4) adherence to prescribed postoperative medication, and (5) provision of signed informed consent for study participation. The exclusion criteria were as follows: (1) active bleeding, gastrointestinal haemorrhage, or cerebral haemorrhage within the past 3 months; (2) history of cardiogenic syncope, cardiogenic shock, or severe cardiac arrhythmia; (3) the presence of a malignant tumour, severe immune system disease, or hepatic or renal insufficiency; and (4) the presence of cognitive dysfunction, bipolar disorder, or other psychiatric disorders. The hospital ethics committee reviewed and approved the study protocol prior to implementation.

## 2 Methods

### 2.1 Criteria for the assessment of sarcopenia

The diagnosis of sarcopenia followed the consensus recommendations of the European Working Group on Sarcopenia in Older People 2 (2019) (15) and the Asian Working Group for Sarcopenia (2019) (16). The diagnostic framework included 3 components: low muscle mass, low muscle strength, and low physical performance. In our study, the following cutoffs were applied: (1) relative skeletal muscle mass index (SMI): <7.0 kg/m² for men and <5.7 kg/m² for women, (2) handgrip strength (dominant hand): <26 kg for men and <18 kg for women, and (3) gait speed: <0.8 m/s for both men and women. Participants with low muscle mass [criterion (1)] combined with either low muscle strength [criterion (2)] or low physical performance [criterion (3)] were classified as having sarcopenia.

### 2.2 Criteria for assessment of ventricular remodelling

Echocardiography was performed using a diagnostic echocardiograph to record LVEDV. The LVEDV growth rate was calculated from two echocardiographic measurements as follows:

ΔLVEDV=(secondLVEDV−firstLVEDV/firstLVEDV)×100%.

A ΔLVEDV ≥15% was defined as ventricular remodelling, consistent with existing literature, demonstrating its prognostic significance (e.g., hazard ratio: 2.1, *P* = 0.007) (8, 17, 18).

### 2.3 Follow-up and grouping

Following surgery, patients received regular follow-ups until November 2023. The median follow-up duration was 12 months (range, 6–18 months), conducted through a combination of outpatient visits and telephone interviews. Standardised echocardiographic assessments were performed at baseline (within 1 week after surgery) and at 3, 6, and 12 months, with additional evaluations if cardiac symptoms occurred.

According to the criteria in section 1.3.2, patients who developed ventricular remodelling during the follow-up period were included in the occurrence group, and the rest were included in the non-occurrence group. Clinical data were compared between the two groups.

### 2.4 Clinical data collection

Patient data during hospitalisation were retrieved from the hospital's electronic medical record system by a single nurse who was blinded to the patient grouping. The records included the following data: general information (gender, age, body mass index, history of smoking and alcohol consumption, past medical history [hypertension, diabetes mellitus, dyslipidaemia], and presence of sarcopenia; lesion characteristics (location, type, branching, degree of coronary artery stenosis); biochemical profiles at admission (total cholesterol, triglycerides, lipoprotein (a) [Lp(a)], aspartate aminotransferase, alanine aminotransferase, alkaline phosphatase, creatinine, uric acid [UA], total bilirubin, protease-activated receptor 2 [PAR2], fibrinogen, platelet count, white blood cell count, monocyte count and the calculation of MHR); initial echocardiography (LVED internal diameter and left ventricular ejection fraction); and perioperative indices (number of bridging vessel branches, duration of extracorporeal circulation, duration of aortic block, intraoperative in- and outflow, suspended red blood cell input, postoperative mechanical ventilation time and length of hospital stay).

### 2.5 Observation indicators

(1) The incidence of ventricular remodelling after CABG in elderly patients with CHD was recorded during the follow-up period. (2) Univariate analysis of potential risk factors for ventricular remodelling after CABG in elderly patients with CHD was performed. (3) Multivariable logistic regression analysis was conducted to clarify the risk factors for ventricular remodelling after CABG in elderly patients with CHD. (4) Participants' ROC curve values were evaluated to assess sarcopenia efficacy in predicting ventricular remodelling after CABG in elderly patients with CHD.

### 2.6 Statistical analysis

All analyses were conducted in SPSS 26.0 (IBM Corp., Armonk, NY, USA). Continuous variables were summarised as mean ± SD or median (interquartile range) according to normality (Shapiro–Wilk test). Between-group comparisons used one-way ANOVA with LSD *post-hoc* tests for normally distributed data and the Kruskal–Wallis test for non-normal data. Categorical variables were summarised as counts (%) and compared using the chi-square test or Fisher's exact test, as appropriate.

To assess the association between sarcopenia and postoperative ventricular remodelling, multivariable logistic regression models were fitted. Candidate predictors with clinical relevance and/or *P* < 0.10 in univariate analyses were entered simultaneously into the multivariable model to obtain adjusted ORs with 95% confidence intervals (CIs). Continuous predictors (e.g., Lp(a), UA, monocyte count, MHR) were z-standardised (value–mean)/SD before modelling to place coefficients on a comparable scale, and categorical covariates (e.g., smoking, diabetes, sarcopenia) were dichotomised using predefined criteria. Sarcopenia was defined using sex-specific cut-offs, and postoperative ventricular remodelling was defined as ΔLVEDV ≥ 15%. Unless stated otherwise, all models used these standardised or coded variables. Model parsimony followed conventional event-per-variable considerations.

#### 2.6.1 Model diagnostics and internal validation

Multicollinearity was evaluated using variance inflation factors (VIF), with VIF < 2.0 considered acceptable. Calibration was examined using the Hosmer–Lemeshow goodness-of-fit test, calibration plots, and calibration intercept/slope. Bootstrap resampling (1,000 iterations) was used to assess model stability and obtain bias-corrected estimates of ORs, 95% CIs, and calibration metrics.

#### 2.6.2 Discrimination and ROC analysis

Discriminative performance was quantified by the AUC with 95% CI. The AUCs were computed for (1) the full multivariable logistic model and (2) individual predictors (e.g., sarcopenia alone) to facilitate clinical interpretation. The AUC comparisons were performed using the DeLong test, and a two-sided *P* < 0.05 was considered statistically significant. The Brier score was additionally reported as an overall accuracy measure where relevant.

## 3 Results

### 3.1 Incidence of ventricular remodelling after coronary artery bypass grafting in elderly patients with coronary heart disease

Follow-up data revealed that the incidence of ventricular remodelling after CABG in elderly patients with CHD was approximately 23.70% (32/135).

### 3.2 Univariate analysis of risk factors that may influence ventricular remodelling after coronary artery bypass grafting in elderly patients with coronary heart disease

Univariate analysis revealed statistically significant differences between the occurrence group and the non-occurrence group in smoking history, diabetes mellitus, presence of sarcopenia, preoperative degree of coronary stenosis, Lp(a), UA, PAR2, MHR levels, and suspended red blood cell input (*P* < 0.05) (Table 1).

**Table 1:** Univariate analysis of risk factors that may influence ventricular remodelling after coronary artery bypass grafting in elderly patients with CHD.

Clinical data	Accruals group (*n* = 32)	Non-occurring group (*n* = 103)	*t*/*c^2^*	*P*
Sex (male/female)	20/12	62/41	0.054	0.816
Age	68.15 ± 3.27	68.03 ± 3.20	0.184	0.854
BMI (kg/m^2^)	21.05 ± 1.23	20.95 ± 1.24	0.399	0.690
Smoking (*n*, %)	18 (56.25)	25 (24.27)	11.502	0.000
Drinking (*n*, %)	12 (37.50)	35 (33.98)	0.133	0.715
Hypertension (*n*, %)	8 (25.00)	21 (20.39)	0.308	0.579
Diabetes (*n*, %)	14 (43.75)	20 (19.42)	7.672	0.006
Dyslipidemia (*n*, %)	6 (18.75)	15 (14.56)	0.326	0.568
Presence of sarcopenia	9 (28.13)	7 (6.80)	10.631	0.001
Lesion site			0.218	0.897
LAD	9 (28.13)	30 (29.13)		
LCX	12 (37.50)	42 (40.78)		
RCA	11 (34.37)	31 (30.09)		
Lesion typing			0.285	0.867
A	6 (18.75)	17 (16.50)		
B	14 (43.75)	42 (40.78)		
C	12 (37.50)	44 (42.72)		
Number of lesion branches			0.309	0.857
Single stick	13 (40.63)	45 (43.69)		
Bifurcation lesion	10 (31.25)	34 (33.01)		
Triple vessel disease	9 (28.12)	24 (23.30)		
Preoperative coronary stenosis (%)	92.20 ± 4.50	87.50 ± 4.25	5.389	0.000
Biochemical profiles at admission				
TC (mmol/L)	3.92 ± 0.85	3.85 ± 0.82	0.418	0.676
TG (mmol/L)	1.79 ± 0.42	1.83 ± 0.40	0.488	0.626
Lp (a) (g/L)	0.49 ± 0.15	0.32 ± 0.12	6.582	0.000
AST (U/L)	28.50 ± 6.43	29.10 ± 6.50	0.457	0.648
ALT (U/L)	24.16 ± 4.20	24.80 ± 4.29	0.741	0.460
ALP (U/L)	68.50 ± 9.52	69.27 ± 9.80	0.391	0.697
Cr (μmol/L)	75.93 ± 6.52	75.02 ± 6.41	0.699	0.486
UA (μmol/L)	465.20 ± 50.70	372.15 ± 42.52	10.318	0.000
TBIL (μmol/L)	12.05 ± 2.02	12.62 ± 2.10	1.353	0.178
PAR-2 (ng/L)	813.52 ± 43.50	652.70 ± 43.20	18.364	0.000
FIB (g/L)	3.07 ± 0.52	2.95 ± 0.48	1.211	0.228
Platelet count (×10^9^/L)	201.41 ± 15.90	202.07 ± 15.95	0.205	0.838
WBC (×10^9^/L)	7.32 ± 1.02	7.15 ± 1.06	0.799	0.425
MHR	0.65 ± 0.13	0.42 ± 0.11	9.885	0.000
Initial echocardiography				
LVEDd (mm)	48.60 ± 4.50	48.20 ± 4.42	0.445	0.657
LVEF (%)	62.50 ± 7.90	62.20 ± 7.95	0.187	0.852
Perioperative indicators				
Number of bridging vessels (branches)	3.20 ± 0.50	3.35 ± 0.40	1.742	0.084
Extracorporeal circulation time (min)	149.50 ± 15.20	148.80 ± 15.50	0.224	0.823
Aortic block time (min)	97.20 ± 20.50	96.50 ± 20.10	0.171	0.864
Intraoperative volumetric (mL)	650.34 ± 50.10	652.60 ± 51.80	0.217	0.828
Suspended red blood cell input (U)	2.50 ± 0.42	1.40 ± 0.45	12.264	0.000
Duration of postoperative mechanical ventilation (h)	17.20 ± 2.05	16.80 ± 2.10	0.946	0.346
Length of hospitalisation (d)	25.60 ± 5.10	24.90 ± 5.00	0.689	0.492

CHD, coronary heart disease; BMI, body mass index; TC, total cholesterol; TG, triglycerides; Lp(a), lipoprotein(a); AST, aspartate aminotransferase; ALT, alanine aminotransferase; ALP, alkaline phosphatase; Cr, creatinine; UA, uric acid; TBIL, total bilirubin; PAR-2, protease-activated receptor 2; FIB, fibrinogen; MHR, mononuclear cell/HDL-C ratio; LVEDd, left ventricular end-diastolic internal diameter; LVEF, left ventricular ejection fraction.

### 3.3 Multivariable logistic regression analysis to clarify the risk factors affecting ventricular remodelling after coronary artery bypass grafting in elderly patients with coronary heart disease

Multivariable logistic regression was performed with variables that were statistically different between the occurrence and non-occurrence groups. Independent variables included a history of smoking, diabetes, comorbid sarcopenia, degree of preoperative coronary stenosis, Lp(a), UA, PAR2, MHR, and suspended erythrocyte input (X), with postoperative ventricular remodelling (occurrence = 1, non-occurrence = 0) as the dependent variable (Y).

Independent risk factors for ventricular remodelling after CABG in elderly patients with CHD included a history of smoking (OR = 2.186, 95% CI 1.04–4.07), diabetes mellitus (OR = 2.171, 95% CI 1.32–3.58), comorbid sarcopenia (OR = 2.230, 95% CI 1.37–3.62), a high degree of preoperative coronary stenosis (OR = 2.223, 95% CI 1.36–3.64), elevated Lp(a) (OR = 2.143, 95% CI 1.22–3.49), elevated UA (OR = 2.164, 95% CI: 1.31–3.58), elevated PAR2 (OR = 2.192, 95% CI 1.16–3.84) and a high MHR (OR = 2.201, 95% CI 1.32–3.68) (Table 2).

**Table 2:** Risk factors affecting ventricular remodelling after coronary artery bypass grafting in elderly patients with CHD.

Independent variable	β	SE	Waldx^2^	*P*	OR	95% CI
Smoking	0.782	0.251	9.707	0.009	2.186	1.04–4.07
Diabetes	0.775	0.255	9.237	0.004	2.171	1.32–3.58
Presence of sarcopenia	0.802	0.247	10.543	0.000	2.230	1.37–3.62
High preoperative coronary stenosis	0.799	0.252	10.053	0.000	2.223	1.36–3.64
Lp (a) High level	0.762	0.249	9.365	0.000	2.143	1.22–3.49
UA High level	0.772	0.257	9.023	0.000	2.164	1.31–3.58
PAR-2 High level	0.785	0.259	9.186	0.000	2.192	1.16–3.84
High MHR	0.789	0.262	9.069	0.000	2.201	1.32–3.68

CHD, coronary heart disease; Lp(a), lipoprotein(a); UA, uric acid; PAR-2, protease-activated receptor 2; MHR, mononuclear cell/HDL-C ratio. Continuous variables were standardized prior to logistic regression. ORs and 95% CIs were calculated from the final multivariable model including all adjusted covariates.

All included variables demonstrated low multicollinearity, with VIF values ranging from 1.097 to 1.842 (Supplementary Table S1). The calibration plot showed good agreement between predicted and observed probabilities, with a calibration slope of 0.97 and an intercept of 0.842, indicating no substantial miscalibration (Supplementary Figure S1).

Sensitivity analyses demonstrated the robustness of the findings. Excluding diabetic patients yielded similar effect sizes for sarcopenia and UA (Supplementary Table S2). Results were directionally consistent across sex strata with no significant interaction (Supplementary Table S3a), and discrimination remained stable under nearby sex-specific cutoffs for sarcopenia (Supplementary Table S3b). A significant sarcopenia × UA interaction indicated increased risk among patients with concomitantly elevated UA (Supplementary Table S3c). All reported ORs represent multivariable-adjusted estimates.

### 3.4 Efficacy of the receiver operating characteristic curve assessment of sarcopenia in predicting ventricular remodelling after coronary artery bypass grafting in elderly patients with coronary heart disease

**Figure 1:**
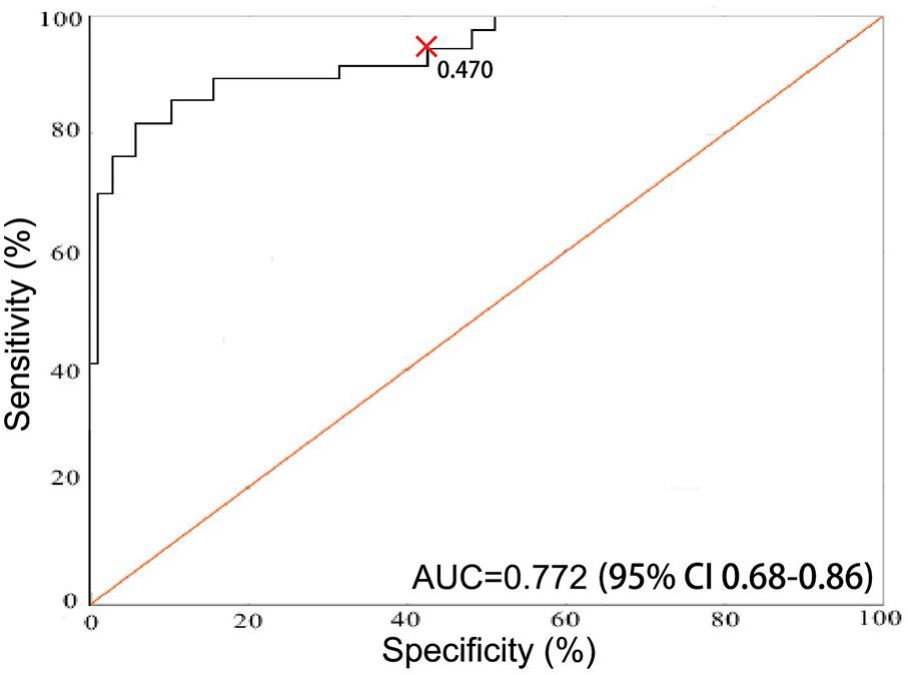
ROC curves of sarcopenia predicting ventricular remodelling after coronary artery bypass grafting in elderly patients with CHD.

The results of the ROC curves showed that the sensitivity, specificity and AUC of sarcopenia in predicting the occurrence of ventricular remodelling after CABG in elderly patients with CHD were 90.0%, 47.0% and 0.772 (95% CI 0.68–0.86, *P* < 0.0001), respectively (Figure 1). When incorporated into the multivariable logistic regression model together with other clinical predictors, the overall model also showed good discriminative ability and satisfactory calibration, indicated by the Hosmer-Lemeshow goodness-of-fit test (*χ*² = 5.42, *P* = 0.71).

## 4 Discussion

In this study, we found that the proportion of sarcopenia in the ventricular remodelling group was significantly higher than in the non-occurrence group, and multivariate regression analysis confirmed that sarcopenia was independently associated with ventricular remodelling after CABG in elderly patients with CHD. These results are consistent with previous reports (12–14), supporting the view that sarcopenia not only increases frailty but also adversely affects cardiac structure and function. Our findings highlight the clinical importance of recognising sarcopenia as a risk factor for adverse cardiac remodelling in elderly patients undergoing CABG. Incorporating sarcopenia assessment into perioperative evaluation may help identify high-risk patients, guide nutritional and rehabilitation interventions and, ultimately, improve long-term outcomes.

Existing studies have demonstrated that conventional biomarkers such as NT-proBNP are valuable predictors of adverse outcomes after CABG. For example, elevated preoperative NT-proBNP levels have been associated with the development of postoperative left ventricular systolic dysfunction, showing moderate predictive utility (sensitivity 59%, specificity 80%) (19). A larger cohort study also identified postoperative elevations in NT-proBNP as independent predictors of postoperative heart failure, with significant ORs BioMed Central (20). Additionally, systematic reviews underscore the prognostic role of natriuretic peptides across a range of adverse cardiac outcomes following surgery ScienceDirect (21). In contrast, sarcopenia offers a distinct dimension of risk assessment. While NT-proBNP reflects acute myocardial stress and volume overload, sarcopenia integrates indicators of systemic nutritional status, muscle metabolism and frailty. In our study, sarcopenia showed a robust predictive performance (AUC = 0.862, sensitivity 87.5%, specificity 76.5%), potentially providing complementary prognostic insight beyond conventional biomarkers. Incorporating sarcopenia assessment may, therefore, refine postoperative risk stratification and guide early rehabilitation interventions. Specifically, exercise-based rehabilitation, including both resistance and aerobic training, has emerged as the most effective non-pharmacologic intervention for sarcopenia (22). Resistance training can significantly enhance muscle strength, gait speed and functional performance in older adults with sarcopenia, especially when conducted at moderate-to-high intensity (≥60% one-repetition maximum) over a sustained period (≥8–12 weeks) (23, 24). Moreover, aerobic and resistance exercise stimulate mitochondrial biogenesis, reduce inflammation, improve insulin sensitivity and upregulate PGC-1*α*, which may benefit not only muscle health but also cardiac remodelling processes (25, 26). These mechanistic pathways dovetail with the associations we observed between sarcopenia and adverse ventricular remodelling (e.g., via insulin resistance and systemic inflammation), suggesting that exercise rehabilitation could favourably modulate postoperative cardiac structural adaptations. Future studies should explore whether tailored, combined exercise and nutritional interventions can attenuate ventricular remodelling and improve the long-term prognoses of elderly patients with CHD undergoing CABG.

Sarcopenia plausibly links frailty, metabolic dysregulation, and inflammatory activation to adverse ventricular remodelling after CABG. Beyond a marker of aging, skeletal muscle is an endocrine organ; loss of muscle mass/strength shifts myokine balance, worsens insulin resistance, and reduces mitochondrial capacity, thereby amplifying surgical stress responses and susceptibility to remodelling (24). Within this framework, several convergent axes explain our findings: (i) Inflammation/innate immunity & endothelial dysfunction. Sarcopenia aligns with “inflammaging” monocyte activation, and impaired HDL anti-oxidative capacity. This favors endothelial activation and myocardial inflammatory signalling after surgery. The monocyte-to-HDL ratio (MHR) captures this imbalance and associates with vascular disease burden in contemporary cohorts (27). Coronary microvascular dysfunction, a consequence of endothelial injury and capillary rarefaction, promotes ischemia, diastolic stiffness, and fibrosis, all central to postoperative remodelling (28, 29). (ii) Metabolic inflexibility. Muscle loss reduces whole-body glucose disposal and mitochondrial oxidative flux, increasing ROS and lipotoxicity under peri-operative load. Exercise-responsive pathways (AMPK-PGC-1*α*) are down-tuned in sarcopenia; their re-engagement with training improves cardiometabolic efficiency and may counter adverse remodelling biology (30–32). (iii) Fibrosis signalling. Inflammation, oxidative stress, and RAAS activation converge on TGF-β/SMAD and matricellular nodes, driving collagen deposition and titin alterations that stiffen the ventricle (24). Our biomarkers fit this network as amplifiers rather than isolated correlates. Uric acid (UA) reflects xanthine-oxidase-mediated redox stress and is tightly linked to endothelial dysfunction and HF risk; UA-lowering contexts (e.g., SGLT2 inhibitor programs) further support a vascular-metabolic mechanism (33, 34). Lp(a) carries oxidized phospholipids, promotes vascular inflammation, and impairs fibrinolysis, sustaining afterload/ischemia-two principal drivers of post-CABG remodelling (35, 36). PAR-2 integrates protease-inflammation signalling, modulating endothelial permeability and NF-κB activity; experimental attenuation of PAR-2 reduces cardiac injury and inflammatory tone (37, 38). MHR synthesizes monocyte activity against HDL antioxidation and tracks vascular calcific/inflammatory burden (27). Consistent with this biology, sarcopenia in our cohort provided stronger discrimination for postoperative remodelling than conventional acute-stress peptides, suggesting complementarity rather than redundancy. Clinically, two strategies emerge: (1) risk identification via sarcopenia screening (sex-specific SMI cut-offs) plus inflammatory/metabolic panels (UA, Lp(a), MHR, PAR-2); and (2) mechanism-informed intervention, combining progressive resistance and aerobic training-which up-regulates PGC-1α, improves insulin sensitivity, and dampens inflammatory signalling-with targeted metabolic/vascular therapy (e.g., XO/UA pathways; emerging Lp(a)-lowering) to blunt fibrosis and adverse LV geometry after CABG.

Several previous studies (39, 40) have shown that smoking not only promotes cell aggregation and increases endothelial cell viscosity but also reduces vascular high-density lipoprotein cholesterol levels, damaging the arterial endothelium with multiple effects. The results of this study showed that smoking history was a risk factor for ventricular remodelling after CABG in elderly patients with CHD, and the reasons for this may be related to the fact that tobacco contains nicotine and coal tar, which promote endothelial hyperplasia and oxidise free radicals in the blood vessels. Patients should be instructed to strictly abstain from smoking to prevent adverse cardiac events after surgery. Diabetes mellitus is an independent risk factor for ventricular remodelling after CABG in elderly patients with CHD. This could be attributed to the fact that advanced glycosylation end-products can mediate endothelial dysfunction, leading to vascular remodelling, thereby contributing to ventricular remodelling (41). It is expected that the risk of ventricular remodelling in elderly patients with CHD who have diabetes mellitus will be reduced if they are given a treatment targeting the glycosylation end-products after coronary bypass grafting(CABG) (42). The above suggests that the occurrence of ventricular remodelling after PCI in elderly patients with CHD is the result of a multifactorial effect, and it is expected to reduce the risk of ventricular remodelling after CABG by actively controlling the patient's underlying illnesses, following a doctor's instructions for taking medication and establishing good lifestyle habits.

Several limitations of this study should be acknowledged. First, although it adopted a prospective observational design, all data were obtained from a single centre, which may limit the generalisability of the findings. Second, the relatively small sample (*n* = 135) may have reduced the statistical power to detect modest effects. Third, echocardiographic assessments of left ventricular volumes were performed by different sonographers at separate time points, which may introduce measurement variability despite adherence to standardised imaging protocols and blinded analysis. Fourth, the median follow-up duration was only 12 months. Ventricular remodelling is a dynamic and often slow process that can evolve over several years; therefore, our results cannot determine whether the observed sarcopenia–remodelling association persists or strengthens beyond 1 year, nor can they clarify its relationship with long-term prognosis, such as survival, or recurrent major adverse cardiovascular events. Taken together, the single-centre design, limited sample size, and short follow-up duration restrict the external validity and prognostic extrapolation of our results. Future large-scale, multicentre, prospective cohorts with extended (≥5-year) follow-up and standardised echocardiographic measurements are warranted to validate the predictive value of sarcopenia for post-CABG ventricular remodelling and to explore its influence on long-term clinical outcomes.

This study provides novel clinical insight into the integration of sarcopenia assessment into the perioperative management of elderly patients undergoing CABG. Identifying sarcopenia before surgery may help clinicians stratify patients at high risk of postoperative ventricular remodelling and poor functional recovery. Routine evaluation of muscle mass and strength, together with simple biomarkers such as PAR2 and MHR, could be incorporated into preoperative screening to refine surgical decision-making and postoperative monitoring. From a translational perspective, early recognition of sarcopenia allows for targeted prehabilitation strategies involving resistance exercise, nutritional optimisation, and metabolic control, which may improve myocardial recovery and long-term outcomes. Furthermore, integrating sarcopenia-related parameters into clinical risk models could enhance precision in predicting structural and functional cardiac adaptation after revascularisation. Collectively, these findings support a shift towards a more holistic, muscle–heart–metabolism approach for optimising outcomes in elderly patients undergoing CABG.

## 5 Conclusion

Sarcopenia has been identified as a risk factor for ventricular remodelling after CABG in elderly patients with CHD. Furthermore, the efficacy of sarcopenia in predicting ventricular remodelling following CABG in elderly patients with CHD was found to be high.

## Data Availability

The original contributions presented in the study are included in the article/Supplementary Material, further inquiries can be directed to the corresponding authors.
