# Supplementary material for: Impact of sarcopenia on ventricular remodelling following coronary artery bypass grafting in elderly patients with coronary heart disease
Source: Front Cardiovasc Med. 2026 Apr 29;12:1635863. doi: 10.3389/fcvm.2025.1635863 (PMC13167404; doi:10.3389/fcvm.2025.1635863)
Supplement: Supplementary file 1 [file Datasheet1.pdf]

Figure S1 Metrics. Model calibration metrics for the penalized logistic model: Brier score, calibration slope/intercept, Hosmer-Lemeshow P.

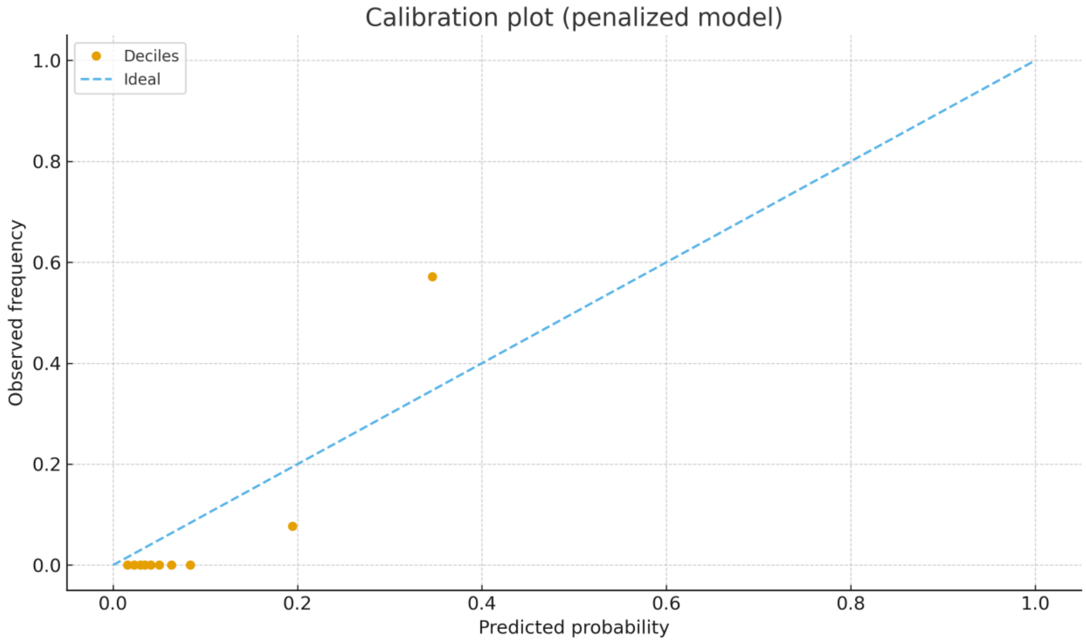

Table S1. Multicollinearity diagnostics of covariates using variance inflation factor (VIF).

Variable	VIF
Smoking	1.508
Diabetes	1.610
Sarcopenia	1.097
LP(a)	1.842
UA	1.703
Monocyte	1.532

Lp(a): lipoprotein(a), UA: uric acid.

Table S2: Sensitivity analysis excluding patients with diabetes mellitus: penalized logistic regression with bootstrap 95% CIs.

Variable	OR	95% CI
Smoking	0.298	0.20-0.45
Diabetes	1	1

Sarcopenia	6.016	2.05-12.00
LP(a)	1.087	0.73-1.72
UA	1.279	0.93-1.85
Monocyte	1.288	0.72-2.08

Lp(a): lipoprotein(a), UA: uric acid.

Table S3a: Sex-stratified associations between predictors and postoperative ventricular remodelling (penalized logistic regression, bootstrap 95% CIs).

Variable	OR	95% CI	Stratum
Smoking	0.647	0.35-1.24	Male
Diabetes	1.119	0.43-2.20	Male
Sarcopenia	3.161	1.16-5.61	Male
LP(a)	1.251	0.72-1.98	Male
UA	1.617	1.03-2.82	Male
Monocyte	1.122	0.61-1.96	Male
Smoking	0.5	0.26-0.91	Female
Diabetes	0.652	0.35-1.13	Female
Sarcopenia	7.384	2.26-13.02	Female
LP(a)	0.922	0.65-1.83	Female
UA	1.376	0.98-2.96	Female
Monocyte	0.979	0.62-1.83	Female

Lp(a): lipoprotein(a), UA: uric acid.

Table S3b: Stratified associations by binary sarcopenia (men <7.0; women <5.7): penalized logistic regression with bootstrap 95% CIs.

Variable	OR	95% CI
smoking	0.537	0.25-1.09
diabetes	0.855	0.37-1.92
LP(a)	1.03	0.59-3.42
UA	3.761	1.66-6.30

Monocyte	1.015	0.49-2.22

Lp(a): lipoprotein(a), UA: uric acid.

Table S3c: Interaction tests: sarcopenia × sex and sarcopenia × uric acid (UA, z-score); bootstrap two-sided P values.

Interaction term	Bootstrap P
Sarcopenia × sex	0.44
Sarcopenia × UA(z)	0.004

UA: uric acid.
